# SMARCB1- and vimentin-positive esophageal carcinoma with undifferentiated components, rhabdoid features, and a good prognosis: a case report

**DOI:** 10.1186/s40792-019-0562-4

**Published:** 2019-01-16

**Authors:** Hideki Nagano, Toshimasa Izumi, Ei Kawahara, Takeru Oyama, Takanori Goi

**Affiliations:** 1 Department of Surgery, Fukui General Hospital, 58-16-1 Egami-cho, Fukui, 910-3113 Japan; 2 Department of Pathology, Fukui General Hospital, 58-16-1 Egami-cho, Fukui, 910-3113 Japan; 3 Department of Pathology, Kanazawa University, 13-1 Takara-machi, Kanazawa, Ishikawa 920-8641 Japan; 4 1st Department of Surgery, Faculty of Medicine, University of Fukui, 23-3 Matsuokashimoaizuki, Yoshida-gun, Eiheiji-cho, Fukui 910-1193 Japan; 5 Department of Surgery, Japan Community Health Care Organization Fukui Katsuyama General Hospital, 2-6-21 Nagayama-cho, Katsuyama, Fukui, Japan

**Keywords:** Carcinoma with undifferentiated components, Rhabdoid features, SMARCB1, Vimentin, CD34, Round cell carcinoma, Esophagus

## Abstract

**Background:**

Undifferentiated carcinoma of the esophagus with rhabdoid features is a very rare histologic finding that is occasionally associated with the loss of SWI/SNF-related matrix-associated actin-dependent regulator of chromatin subfamily B member 1 (SMARCB1); however, until now, few survey reports of this type of tumor have been published. In this study, we describe a case of esophageal carcinoma with undifferentiated components and rhabdoid features that was exclusively positive for vimentin and SMARCB1 in a patient with prolonged survival.

**Case presentation:**

A 67-year-old man complained of a stomachache and loss of appetite persisting for 1 month. He was then admitted to the hospital. Diagnostic imaging studies revealed a transdiaphragmatic circular ulcerative tumor of the esophagogastric region. Biopsy specimens showed undifferentiated round cell carcinoma. The patient underwent lower esophageal resection and total gastrectomy with lymph node dissection. Microscopic analysis revealed that most of the primary tumor consisted of large undifferentiated round cells and scattered rhabdoid cells. The tumor invaded the muscular layer in the esophagus and the subserosal layer in the stomach, and metastasis was noted in only one lymph node. Immunohistochemical analysis revealed that the round and rhabdoid cells found in the primary tumor were diffusely positive for SMARCB1 and vimentin. The tumor displayed focal positivity for the anti-pan-cytokeratin antibody AE1/AE3. In the positive lymph node, round undifferentiated carcinoma cells were admixed with squamous carcinoma cells that were positive for cytokeratin 5/6 and 34βE12. The MIB-1 index was 19.7% and 0.5% for the round cells from the primary tumor and epithelial cells from the metastatic lymph node lesion, respectively, and 70.1% for the round cells from the metastatic lymph node lesion. The patient has been alive for 10 years after surgery without tumor recurrence.

**Conclusions:**

We reported a rare case of esophageal carcinoma with undifferentiated components, rhabdoid features, and a good prognosis.

## Background

Rare undifferentiated carcinoma of the esophagus, which is an aggressive neoplasm that is associated with a high incidence of recurrence and/or metastases and a dismal prognosis [1], is characterized by polypoid or sheet-like growth of undifferentiated tumor cells [2]. This malignancy has also been referred to as pseudosarcoma, carcinosarcoma, or sarcomatoid carcinoma, which is usually admixed with squamous cell carcinoma (SCC) [1, 3, 4] and is sometimes accompanied by a chondroid matrix [5]. Previously, round cell subtypes of undifferentiated carcinoma were differentially subclassified; one of such subclass with neuroendocrine granules has been identified as neuroendocrine carcinoma [6, 7], and lymphocyte-rich undifferentiated carcinoma is referred to as lymphoepithelial carcinoma [8, 9] and is associated with Epstein-Barr virus (EBV) infection [10]. Recently, the round cell subtype of undifferentiated esophageal carcinoma with prominent rhabdoid features has been identified as a distinct aggressive type of malignancy [11–14], since the tumor is occasionally negative for one of the tumor-suppressor gene products, SWI/SNF-related matrix-associated actin-dependent regulator of chromatin subfamily B member 1 (SMARCB1) [14]. SMARCB1 deficiency is used to define malignant rhabdoid tumors (MRTs) and some carcinomas with rhabdoid features [15–17]. Vimentin-positive gastric carcinomas with rhabdoid features are also known to have a poor prognosis [18], although the prognosis of vimentin-positive carcinoma is still unknown [19].

Thus, undifferentiated round cell carcinomas can be subdivided into several types with distinct immunohistochemical features. However, whether there are undifferentiated round cell carcinomas without distinct immunohistochemical features other than diffuse vimentin positivity is unknown. In this study, we describe a case of esophageal carcinoma with undifferentiated components and rhabdoid features that was exclusively positive for vimentin and SMARCB1 and associated with prolonged survival.

## Case presentation

**Fig. 1:**
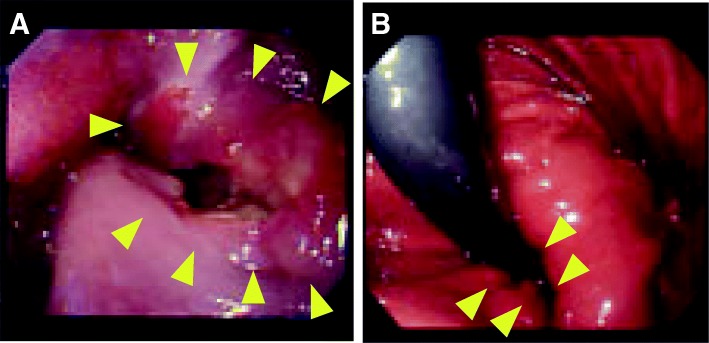
Endoscopic findings. An ulcerated tumor located in the lower esophagus (**a**) and extending to the upper stomach. The inferior border of the tumor was located in the gastric mucosa in a hiatal hernia (**b**)

**Fig. 2:**
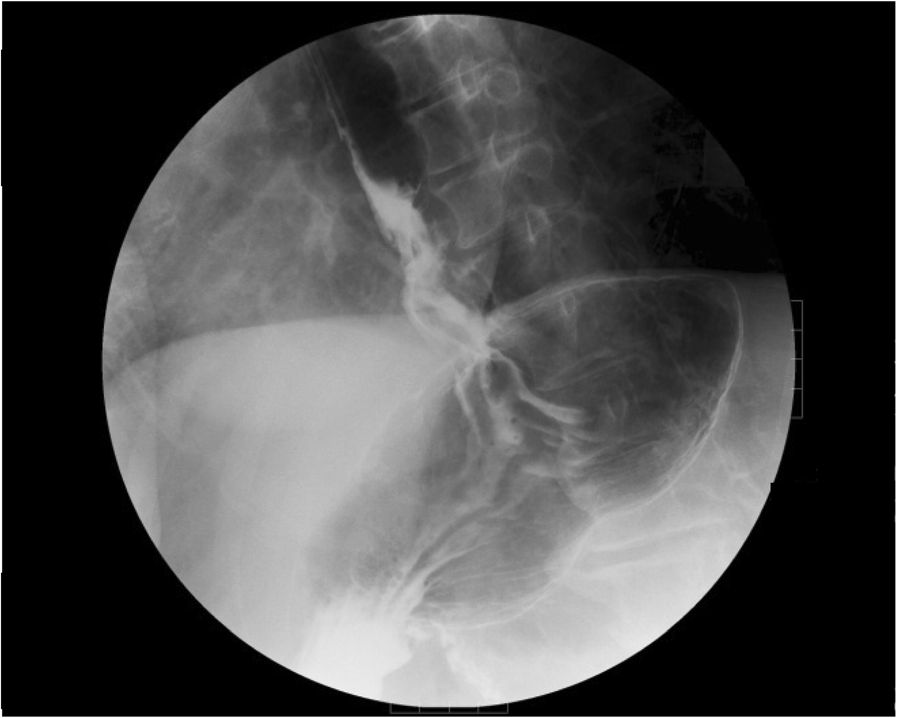
Upper gastrointestinal (GI) fluoroscopy showed a transdiaphragmatic, circular ulcerative tumor that measured 7 cm along its major axis within the esophageal hiatus

A 67-year-old man complained of a stomachache and loss of appetite persisting for more than 1 month and was admitted to Fukui General Hospital. The patient’s medical and family history were unremarkable. The patient smoked ten cigarettes a day from the age of 20 to 67 years. The patient also reported heavy alcohol consumption for 10 years or longer but had stopped drinking. A physical examination revealed no anemia, edema, or malnutrition. Additionally, there were no abnormalities in his laboratory data including levels of tumor markers such as carcinoembryonic antigen (CEA), carbohydrate antigen 19-9 (CA19-9), SCC antigen, and CA125. Endoscopic examination revealed an ulcerated tumor in the lower esophagus, 33 cm from the incisors (Fig. 1). The tumor extended from the lower esophagus to the upper part of the stomach. Biopsy specimens showed poorly differentiated carcinoma without any features of differentiation, suggesting poorly differentiated SCC or undifferentiated carcinoma. Upper gastrointestinal fluoroscopy revealed a transdiaphragmatic, circular ulcerative tumor that measured 7 cm along its major axis (Fig. 2). Enhanced computed tomography (CT) showed a swollen lymph node along the left paracardiac region and the left gastric artery. No distant metastasis was detected. According to these diagnostic imaging findings, a preoperative clinical diagnosis of T3N1M0 stage III cancer was made using the Union for International Cancer Control (UICC) classification system.

The patient underwent a lower esophageal resection and total gastrectomy with lymph node dissection in December 2008. He had an uneventful recovery. Adjuvant chemotherapy consisted of three courses of 5-fluorouracil (5FU) plus cis-diamminedichloroplatinum (CDDP) and oral tegafur-uracil (UFT) for 1 year following surgery. The patient did not show recurrence for 10 years.

## Pathological findings

**Fig. 3:**
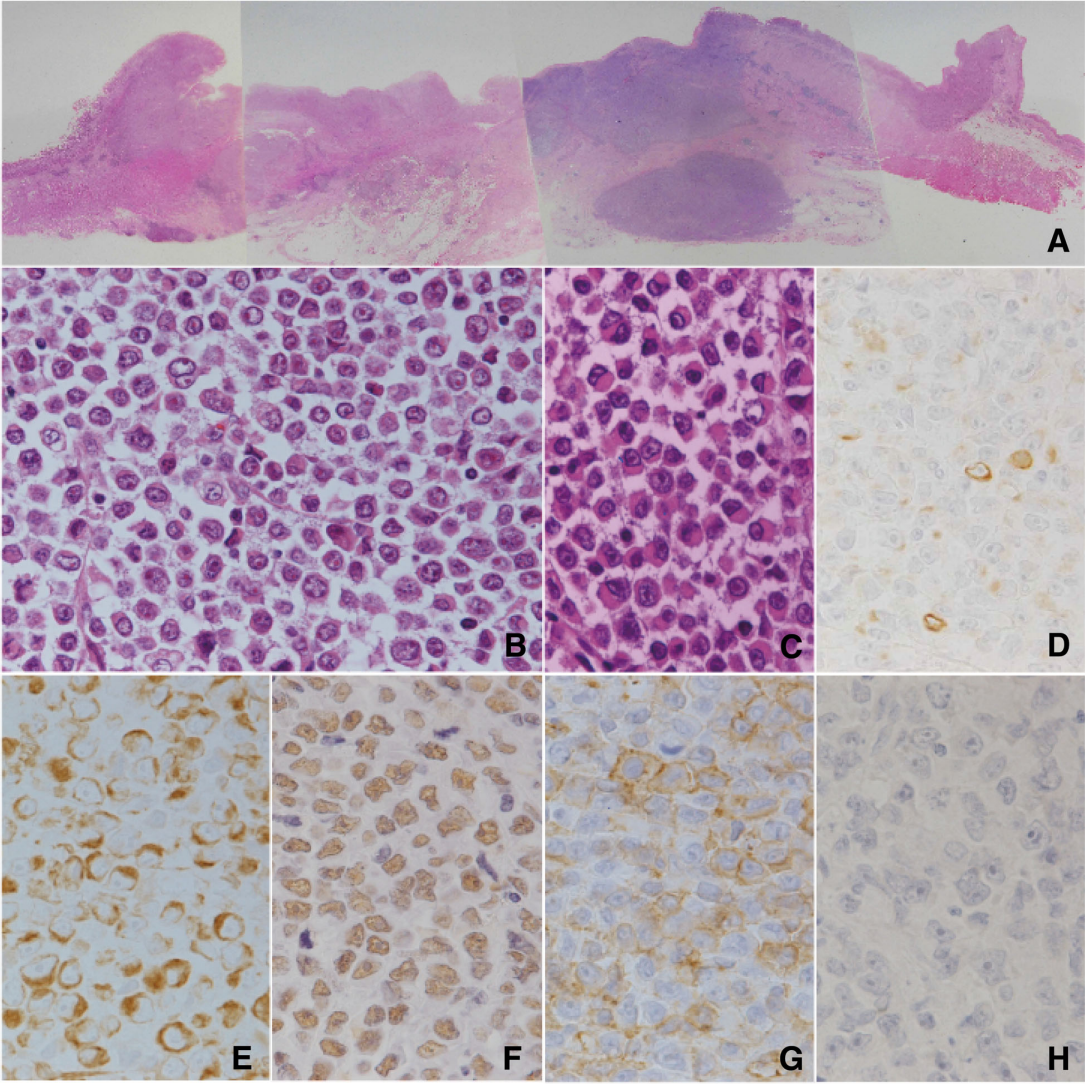
Pathological findings of the resected primary tumor. A loupe image of the primary gastroesophageal region (**a** HE) shows that the tumor invades the muscular layer in the esophagus and the subserosal layer in the stomach. In the primary tumor, large uniform round-shaped cells with relatively little cytoplasm were arranged in a diffuse pattern (**b** HE, × 400), and some of the round cells showed prominent features of rhabdoid cells (**c** HE, × 400). The round tumor cells were prominently vimentin positive (**e** × 400) and weakly positive for cytokeratin (AE1/AE3) (**d** × 400). The cells were also positive for SMARCB1 (**f** × 400). The undifferentiated cells were positive for CD34 (**g** × 400), but negative for c-kit (**h** × 400)

A polypoid tumor with ulceration measuring 7.5 × 5.5 cm in size was found in the gastroesophageal region (Fig. 3a). The tumor invaded the muscular layer of the esophagus and the subserosal layer of the stomach. Microscopic analysis revealed that most of the tumor consisted of large round-shaped cells with scant cytoplasm and little cell-to-cell contact (Fig. 3b). The cells had large nuclei and prominent nucleoli. Features of differentiation, including gland formation, mucin production, and keratinization, were not found. Immunohistochemical antibodies used for tumor diagnosis are listed in Table 1.

**Table 1:** Immunohistochemical antibodies used for tumor diagnosis

IHC marker	Dilution	Company	Antibody
Pan-cytokeratin	Ready to use	Dako	AE1/AE3
Low molecular cytokeratin	Ready to use	Becton Dickinson	CAM5.2
High molecular cytokeratin	1:50	Dako	34βE12
CK 5/6	Ready to use	Dako	D5/16 B4
CK 7	Ready to use	Dako	OV-TL 12/30
CK 20	Ready to use	Dako	Ks 20.8
Vimentin	Ready to use	Dako	V9
Myoglobin	Ready to use	Roche	Polyclonal
CD 30	Ready to use	Dako	Ber-H2
CD 34	Ready to use	Dako	QBEnd 10
LCA	Ready to use	Dako	PD7/26 and 2B11
EMA	Ready to use	Dako	E29
CA 19-9	1:50	Dako	1116-NS-19-9
S 100	Ready to use	Dako	Polyclonal
c-kit	1:400	Dako	Polyclonal
LMP-1	1:200	Dako	CS.1-4
Chromogranin A	Ready to use	Dako	Polyclonal
Synaptophysin	Ready to use	Nichirei	27G12
CD56	Ready to use	Nichirei	MRQ-42
SMARCB1	1:200	Abnova	BAF47

Focally, the round-shaped cells with rhabdoid features, polygonal morphology (Fig. 3c), and abundant eosinophilic cytoplasm were scattered. An epithelial component was not observed in the primary tumor. The tumor invaded the muscularis propria in the esophageal part and reached the subserosal layer in the gastric part. Lymphatic permeation was not found, and venous involvement was detected.

**Fig. 4:**
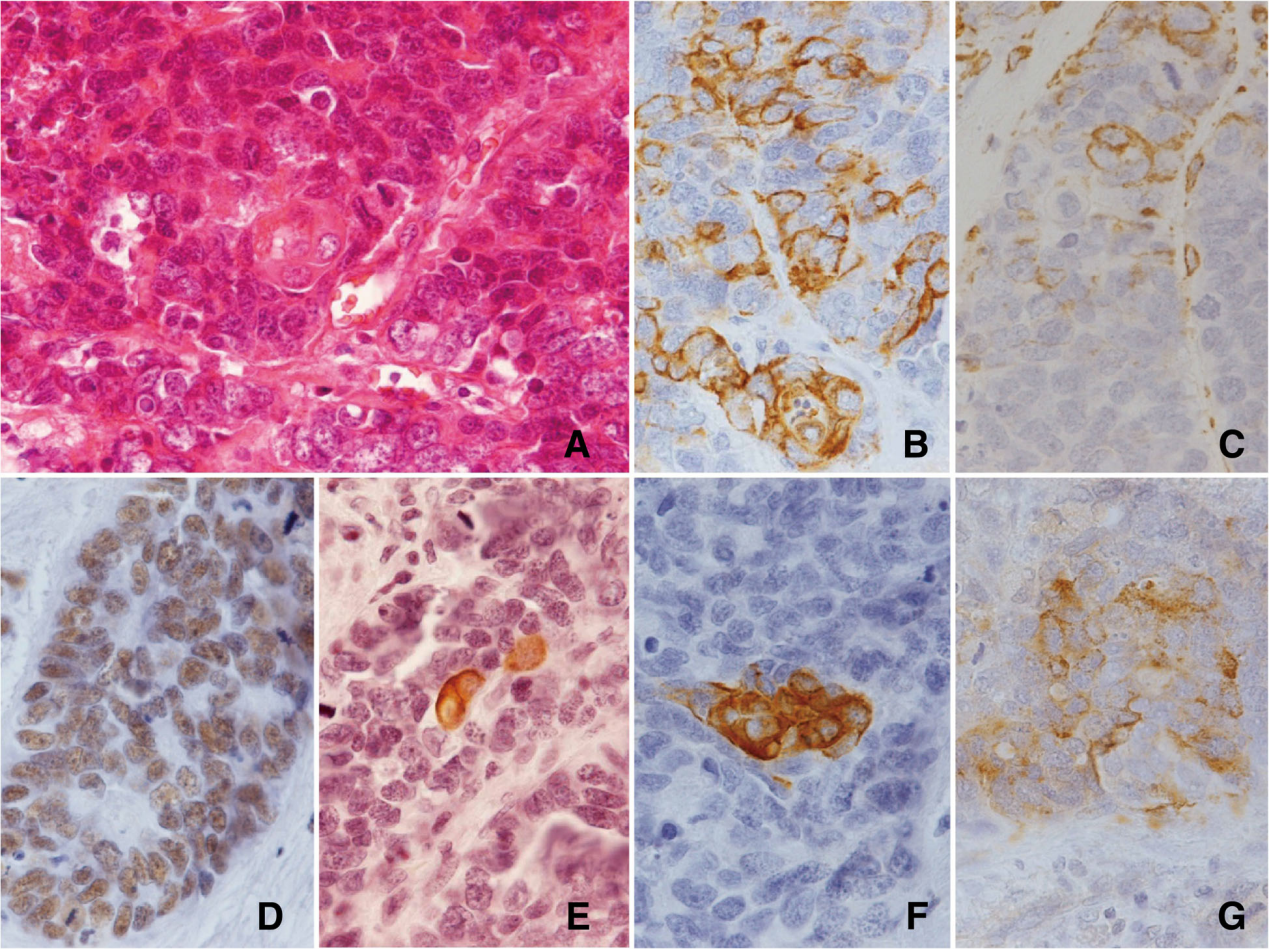
Pathological findings of squamous differentiation in the lymph node (**a** HE, × 400). The carcinoma cells were positive for AE1/AE3 antibodies (**b** × 400) and focally positive for vimentin (**c** × 400). The carcinoma cells were exclusively positive for SMARCB1 (**d**; × 400). The squamous carcinoma differentiation was positive for cytokeratin 5/6 (**e**; × 400) and anti-high molecular cytokeratin antibodies, clone 34βE12 (**f** × 400). The SCC component was positive for chromogranin A (**g** × 400)

Metastasis was detected in a lymph node located along the left paracardiac region. In the metastatic area, foci of carcinomatous components with epithelial cell connections were scattered. Gland formation and/or mucin production was not found. In the foci of the epithelial component, which consisted of cohesive polygonal cells with scant cytoplasm, small clusters of a few polygonal cells with broad eosinophilic cytoplasm suggested that squamous cell differentiation was present (Fig. 4a). A postoperative pathological diagnosis of T3N1M0 stage III cancer was made according to the UICC classification system.

The results of the immunohistochemical analysis are listed in Table 2. The immunohistochemical analysis revealed that the round cells of the primary lesion were diffusely positive for vimentin, SMARCB1, and CD34 (Fig. 3e–g). A few cells in the primary lesion were positive for antibodies against a broad spectrum of cytokeratins including AE1/AE3 (Fig. 3d) and CAM5.2. The cells were negative for antibodies against a restricted spectrum of cytokeratins including CK5/6, 34βE12, CK7, and CK20. CD30, c-kit (Fig. 3h), epithelial membrane antigens (EMAs), CA19-9, chromogranin A, synaptophysin, CD56, myoglobin, leukocyte common antigens (LCAs), S100 protein, and EBV latent membrane protein 1 (LMP-1) were also negative. EBV-encoded small RNA (EBER) was not detected with in situ hybridization.

**Table 2:** Results for immunohistochemistry

	Primary Round cell	Lymph Node Metastasis	
		Round cell	Epithelial
AE1/AE3	−	+ (focal)	++
CAM5.2	−	−	++
34βE12	−	−	+(focal)
CK5/6	−	−	+(focal)
CK7	−	−	−
CK20	−	−	−
Vimentin	++	++	+ (focal)
Myoglobin	−	−	−
CD30	−	−	−
CD34	++	++	++
LCA	−	−	−
EMA	−	−	++
CA19-9	−	−	−
S100	−	−	−
c-kit	−	−	−
LMP-1	−	−	−
Chromogranin A	−	−	+(focal)
Synaptophysin	−	−	+(focal)
CD56	−	−	+
SMARCB1	++	++	++
MIB1 index	19.7%	70.1%	0.5%

*EMA* epithelial membrane antigen, *LCA* leukocyte common antigen

The metastatic carcinoma showing epithelial connections was diffusely positive for AE1/AE3 (Fig. 4b), CAM 5.2, EMA, and SMARCB1 (Fig. 4d). Cells with squamous cell differentiation were positive for CK5/6 and 34βE12 (Fig. 4e, f) and negative for CK7 and CK20, providing immunohistochemical support for squamous cell differentiation. Vimentin-positive carcinoma cells (Fig. 4c) were randomly scattered among the cells with epithelial connections. Chromogranin A (Fig. 4g), synaptophysin, and CD56 were focally positive. The MIB-1 index was 19.7% and 0.5% for the round cells from the primary tumor and epithelial cells from the metastatic lymph node lesion, respectively, and 70.1% for the round cells in the metastatic lymph node lesion.

## Discussion

The present case revealed complete dedifferentiation of the tumor in the esophagus in the largest examined split face of the resected specimen, although the metastatic tumor cells showed differentiation that suggested the possible origin of SCC in a lymph node. The primary round cell undifferentiated carcinoma tissue was exclusively vimentin positive with partially rhabdoid features mimicking rhabdoid carcinoma. The MRT showed loss of the SMARCB1 gene. The SMARCB1 immunohistochemical analysis is a very sensitive tool for diagnosing MRT and some carcinomas with rhabdoid features [13, 20]. Most MRTs are characterized by the loss of SMARCB1; however, carcinomas with rhabdoid features do not always involve the loss of SMARCB1. Agaimy et al. [13] reviewed 39 cases of carcinomas of the digestive tract with rhabdoid features, and death occurred in these patients regardless of the expression status of SMARCB1 or the presence or absence of an epithelial component. The mechanisms responsible for this morphological and biological aggressive shift in SMARCB1 expression remain unknown. However, Agaimy et al. showed heterogeneous subgroups among carcinomas with rhabdoid features in the digestive tract. The tumor in the present case mimicked rhabdoid carcinoma with its rhabdoid features and showed a positive expression of SMARCB1. The round cell carcinoma component also did not show neuroendocrine differentiation, although the squamous cell components in the lymph node showed small foci of neuroendocrine differentiation. Neuroendocrine granules are frequently positive in SCC [21], and the present case could not be regarded as neuroendocrine carcinoma. Lymphocytic infiltration was not marked as reported in lymphoepithelial carcinoma [7, 8, 16, 17], and the negativity for EBER and LMP-1 ruled out EBV-related lymphoepithelial carcinoma. Furthermore, CD34 expression, which has not been reported in gastrointestinal carcinomas, was positive, which might suggest true dedifferentiation since it has been reported that CD34 is a general marker of progenitor cells [22]. Undifferentiated round cell carcinomas that do not show specific features have not been well described, with a few exceptions [23].

We diagnosed the present case as esophageal carcinoma with undifferentiated components and rhabdoid features because we were unable to prove that the patient did not have SCC or undifferentiated carcinoma based on the limited number of specimens that we examined. Although it remains unknown whether there was SCC in the primary tumor, the presence of SCC components in the lymph node metastasis suggests the likelihood of SCC in the primary lesion; therefore, it is possible that the diagnosis of undifferentiated carcinoma with rhabdoid features was inaccurate.

Undifferentiated carcinomas are rare types of esophageal tumors. These tumors have been reported to have aggressive biological behavior and poor prognosis; however, sarcomatoid has been shown to have a better prognosis than SCC of the esophagus, with an overall survival rate of 50% for sarcomatoid carcinomas compared with 3- and 5-year survival rates of 29.8% and 15%, respectively, for SCC [24]. Undifferentiated carcinomas should be subclassified since the prognosis could be different among the different types. Classical round cell carcinomas, including neuroendocrine carcinoma and carcinoma with rhabdoid features, reportedly have a poor prognosis. Considering the preoperative diagnosis of advanced undifferentiated lower esophageal cancer, we feared the possibility of a more aggressive lymphatic permeation or a more extensive lymph node metastases than that observed in ordinal esophageal cancer, and we did not perform a proximal gastrectomy but rather performed a total gastrectomy with lower esophageal resection and lymph node dissection without evidence of positivity.

Conversely, several reports of lymphoepithelioma-like carcinomas of the esophagus have shown these tumors to have a good prognosis [25–30]. However, only a few [10, 30] of 20 or more cases reported were positive for EBER. Then, undifferentiated round cell carcinoma with lymphocytic infiltration, to some extent, might have been misclassified as lymphoepithelioma-like carcinoma since lymphoepithelial carcinoma is defined as undifferentiated carcinoma with abundant lymphoid stroma [31]. Taken together, undifferentiated round cell carcinomas should be subclassified because the prognosis and etiology of these tumors could be different.

## Conclusions

We reported a rare case of esophageal carcinoma with undifferentiated components, rhabdoid features, and a good prognosis.

## Abbreviations

5FU: 5-Fluorouracil
CA125: Carbohydrate antigen 125
CA19-9: Carbohydrate antigen 19-9
CDDP: Cis-diamminedichloroplatinum
CEA: Carcinoembryonic antigen
CT: Computed tomography
EBER: Epstein-Barr virus encoded small RNA
EBV: Epstein-Barr virus
EMA: Epithelial membrane antigen
HE: Hematoxylin-eosin
LCA: Leukocyte common antigen
LMP-1: Epstein-Barr virus latent membrane protein 1
MRT: Malignant rhabdoid tumor
SCC: Squamous cell carcinoma
SMARCB1: SWI/SNF-related matrix-associated actin-dependent regulator of chromatin subfamily B member 1
UFT: Tegafur-uracil
UICC: Union for International Cancer Control
